# Single-Nucleus RNA Sequencing Reveals Cellular Transcriptome Features at Different Growth Stages in Porcine Skeletal Muscle

**DOI:** 10.3390/cells14010037

**Published:** 2025-01-02

**Authors:** Ziyu Chen, Xiaoqian Wu, Dongbin Zheng, Yuling Wang, Jie Chai, Tinghuan Zhang, Pingxian Wu, Minghong Wei, Ting Zhou, Keren Long, Mingzhou Li, Long Jin, Li Chen

**Affiliations:** 1 Chongqing Academy of Animal Science, Chongqing 402460, China; 2 State Key Laboratory of Swine and Poultry Breeding Industry, College of Animal Science and Technology, Sichuan Agricultural University, Chengdu 625041, China; 3 National Center of Technology Innovation for Pigs, Chongqing 402460, China; 4 Sichuan Litian Animal Husbandry Co., Ltd., Dazhou 635711, China; 5 Key Laboratory of Agricultural Bioinformatics, Ministry of Education, Chengdu 625041, China; 6 Key Laboratory of Animal Resource Evaluation and Utilization (Pigs), Ministry of Agriculture and Rural Affairs, Chongqing 402460, China

**Keywords:** skeletal muscle, pig, single-nucleus RNA sequencing, development, IMF

## Abstract

Porcine latissimus dorsi muscle (LDM) is a crucial source of pork products. Meat quality indicators, such as the proportion of muscle fibers and intramuscular fat (IMF) deposition, vary during the growth and development of pigs. Numerous studies have highlighted the heterogeneous nature of skeletal muscle, with phenotypic differences reflecting variations in cellular composition and transcriptional profiles. This study investigates the cellular-level transcriptional characteristics of LDM in large white pigs at two growth stages (170 days vs. 245 days) using single-nucleus RNA sequencing (snRNA-seq). We identified 56,072 cells across 12 clusters, including myofibers, fibro/adipogenic progenitor (FAP) cells, muscle satellite cells (MUSCs), and other resident cell types. The same cell types were present in the LDM at both growth stages, but their proportions and states differed. A higher proportion of FAPs was observed in the skeletal muscle of 245-day-old pigs. Additionally, these cells exhibited more active communication with other cell types compared to 170-day-old pigs. For instance, more interactions were found between FAPs and pericytes or endothelial cells in 245-day-old pigs, including collagen and integrin family signaling. Three subclasses of FAPs was identified, comprising FAPs_*COL3A1*^+^, FAPs_*PDE4D*^+^, and FAPs_*EBF1*^+^, while adipocytes were categorized into Ad_*PDE4D*^+^ and Ad_*DGAT2*^+^ subclasses. The proportions of these subclasses differed between the two age groups. We also constructed differentiation trajectories for FAPs and adipocytes, revealing that FAPs in 245-day-old pigs differentiated more toward fibrosis, a characteristic reminiscent of the high prevalence of skeletal muscle fibrosis in aging humans. Furthermore, the Ad_*PDE4D*^+^ adipocyte subclass, predominant in 245-day-old pigs, originated from FAPs_*PDE4D*^+^ expressing the same gene, while the Ad_*DGAT2*^+^ subclass stemmed from FAPs_*EBF1*^+^. In conclusion, our study elucidates transcriptional differences in skeletal muscle between two growth stages of pigs and provides insights into mechanisms relevant to pork meat quality and skeletal muscle diseases.

## 1. Introduction

Pork is a crucial agricultural product and a major source of protein in human diets. It has also gained prominence as a model organism in biomedical research due to its anatomical and physiological similarities to humans. Skeletal muscle, the primary component of pork, undergoes processes such as cell differentiation and energy regulation during growth that influence muscle fiber composition and intramuscular fat (IMF) content. Consequently, studying skeletal muscle in pigs at different growth stages can offer valuable insights into meat quality improvement and the exploration of disease mechanisms.

Recent studies have begun to unravel the molecular mechanisms governing pig skeletal muscle. For example, RNA sequencing has provided insights into the developmental characteristics of skeletal muscle across different regions and the transcriptional differences among muscle fiber types [1,2,3]. Additionally, integrating ATAC-seq and RNA-seq has advanced our understanding of genes like *ASNS* and *CARNS1*, which regulate muscle development through classical pathways such as Wnt and mTOR signaling [4]. Key genes like *ACBD7*, *TMEM220*, and *ATP1A2* have also been identified, along with *cis*-regulatory elements containing transcription factor binding sites for factors such as SP1 and EGR1, which regulate *ACBD7* and *TMEM220* [5]. While bulk RNA-seq has proven effective for observing overall skeletal muscle characteristics, it does not provide sufficient resolution to reveal transcriptional changes at the cellular level, given the complexity and dynamic regulation of muscle growth.

Single-cell RNA sequencing (scRNA-seq) offers a powerful tool for investigating tissues at the cellular level. In the context of pig skeletal muscle, scRNA-seq has elucidated interactions between muscle satellite cells and FAPs, mediated by the *FGF7–FGFR2* axis, where *FGF7* promotes MUSC proliferation and delays aging [6]. Studies of myogenic differentiation have also identified key regulatory factors like *EGR1* and *RHOB* involved in embryonic myogenesis [7]. Although scRNA-seq has yielded promising results, single-nucleus RNA sequencing (snRNA-seq) is better suited for studying muscle fibers containing multiple nuclei [8]. Recent work integrating snRNA-seq and bulk RNA-seq has identified three subpopulations of adipocytes (*PDE4D*/*PDE7B*, *DGAT2*/*SCD*, and *FABP5*/*SIAH1* cells) and examined the transition of FAPs to IMF cells, providing new insights into the molecular characteristics of marbled muscle [9].

Most studies to date have focused on prenatal or early postnatal stages of skeletal muscle growth, but growth continues into later stages, accompanied by significant increases in IMF content. In humans, the accumulation of IMF with age is often linked to skeletal muscle diseases such as sarcopenia [10]. IMF deposition in humans manifests as a pathologic accumulation of fat in skeletal muscle and is considered a metabolic disorder mediating muscle atrophy and muscle dysfunction [11]. However, in animal production, skeletal muscle fat content is usually positively correlated with meat quality, and high IMF improves meat quality parameters, including meat color, tenderness, and juiciness. Research has also shown that pig and human skeletal muscles share similar cellular components and fat infiltration mechanisms in older age [12]. However, the transcriptional changes at the cellular level during the later growth stages of pig skeletal muscle remain poorly understood.

To further investigate the molecular mechanisms underlying skeletal muscle growth, this study employs snRNA-seq to analyze the transcriptomes of LDM from pigs at 170 and 245 days. This analysis explores the differences in transcription, metabolism, and differentiation of skeletal muscle cells at these two growth stages.

## 2. Materials and Methods

### 2.1. Nuclei Isolation from Skeletal Muscle

Skeletal muscle tissue was obtained from the latissimus dorsi muscle (between the orthogonal 6–7 ribs and the penultimate 3–4 ribs) of 170-day-old and 245-day-old large white pigs. The samples were washed in pre-cooled PBS buffer containing 2 mM EGTA (PBSE). Nuclei isolation was performed using the GEXSCOPE^®^ Nucleus Separation Solution (Singleron Biotechnologies, Nanjing, China), following the manufacturer’s protocol. The isolated nuclei were resuspended in PBSE to a concentration of 10^6^ nuclei per 400 μL, filtered through a 40 μm cell strainer, and counted using Trypan blue staining. Nuclei were defined as DAPI-positive singlets.

### 2.2. Determination of Intramuscular Fat Content

Intramuscular fat content was determined using the Soxhlet extraction method. The intramuscular fat content was calculated using the formula: (Weight data after 4 h of baking—weight data after 40 min of baking on the machine)/net weight of sample powder ∗ 100 = intramuscular fat value. For intramuscular fat staining, fixed fresh tissue was dehydrated in a 15% sucrose solution and at 4 °C. Subsequently, the tissue was embedded in OCT medium and rapidly frozen. Frozen tissues were stained with Oil Red O for 8–10 min, followed by background differentiation with 60% isopropyl alcohol and rinsing with plain water. Hematoxylin staining was performed for 3–5 min, and sections were differentiated in 60% ethanol, rinsed with distilled water, and then blued with bluing reagent. Sections were mounted with glycerol gelatin and viewed under a microscope; the fat droplets were orange to bright red, and the nuclei were blue.

### 2.3. Hematoxylin and Eosin Staining

Paraffin sections were sequentially immersed in xylene I for 20 min, xylene II for 20 min, 100% ethanol I for 5 min, 100% ethanol II for 5 min, 75% and ethanol for 5 min, and then de-paraffinized and finally rinsed in distilled water. Hematoxylin staining was performed for 3–5 min, followed by washing and dehydration for 5 min for eosin staining. The sections were then dehydrated in 100% ethanol (I, II, and III), washed in xylene I and II, and mounted with neutral balsam. For microscopic analysis, images were captured using a digital pathology scanner, with nuclei shown in blue and cytoplasm in red.

### 2.4. Single Nucleus RNA-Sequencing Library Preparation

The concentration of the single-nucleus suspension was adjusted to 3–4 × 10^5^ nuclei/mL in PBS. The suspension was loaded onto a microfluidic chip (GEXSCOPE^®^ Single Nucleus RNA-seq Kit, Singleron Biotechnologies), and snRNA-seq libraries were constructed according to the manufacturer’s instructions. The resulting libraries were sequenced on a DNBSEQ-T7 instrument, generating 150 bp paired-end reads.

### 2.5. Single-Nucleus RNA-Seq Data Processing

Raw reads were mapped to the *Sus scrofa* reference genome (Ensembl version 110) using Celescope (version 1.17.0). The number of unique molecular identifiers (UMIs) per gene per cell was estimated by counting reads associated with the same cell barcode and UMI. UMI count tables for each cell barcode were imported for downstream analysis. SoupX (version 1.6.2) was used to remove low levels of ambient RNA contamination [13], and cells with high proportions of putative doublets were identified using DoubletFinder (version 2.0.3) and subsequently discarded [14].

Cell type clustering and identification were performed using the single-cell analysis software Scanpy (version 1.9.3) [15]. Nuclei with UMI counts between 200 and 10,000, gene counts between 200 and 4000, and mitochondrial gene counts of less than 5% were retained for further analysis. After data normalization, principal component analysis (PCA) was performed to reduce dimensionality, and Harmony was used to remove batch effects. Cells were classified through an unsupervised clustering process, and the *rank_genes_groups* function and Wilcoxon rank-sum test were employed to identify highly differential genes within each cell cluster.

### 2.6. Functional Enrichment Analysis

Pathway enrichment analysis was conducted using Metascape, a web-based tool that integrates multiple gene annotation and analysis resources [16]. During the analysis, pig gene IDs were converted to their human homologs, and gene enrichment in Gene Ontology (GO) and Kyoto Encyclopedia of Genes and Genomes (KEGG) pathways was calculated and visualized. Pathways with *p*-values < 0.01 were considered significant.

### 2.7. Single-Cell Regulatory Network Inference

Regulatory networks in single-nucleus data were analyzed using the pySCENIC-based framework (version 0.12.1) [17]. Pig genes were converted to human homologs based on Ensembl annotations, retaining only one-to-one homologous genes. Regulatory networks were predicted using GRNBoost2 based on co-expression between regulators and targets. CisTarget was then applied to exclude indirect targets and to identify transcription factor (TF) binding motifs. AUCell was used to quantify the regulon activity in each cell. The regulon specificity scores (RSS) were computed to assess the uniqueness of TF regulatory networks in different cell types. The connectivity specificity index (CSI), calculated based on correlations between regulon regulatory networks, was used to assess the strength of interactions [18]. Unsupervised hierarchical clustering based on Euclidean distance was performed to identify distinct regulon modules.

### 2.8. Cell–Cell Interaction Analysis

Cell–cell interactions were analyzed using CellPhoneDB (version 5.0.0) [19], a publicly available database of ligand–receptor interactions. Potential ligand–receptor interactions were inferred based on receptor expression in one cell lineage and ligand expression in another. Only ligands and receptors expressed in more than 10% of cells in a given lineage were considered. To assess lineage specificity, labels for all cells were permuted 1000 times, and the mean ligand–receptor expression across interacting lineages was calculated. A null distribution was generated for each ligand–receptor pair in pairwise comparisons between lineages, and *p*-values for lineage specificity were obtained. Ligand–receptor pairs with *p*-values < 0.05 were considered statistically significant.

### 2.9. Pseudotime Analysis

The developmental and differentiation trajectories of cells of interest were analyzed using Monocle3 (version 1.3.4) [20]. Pseudotime analysis was performed using the default parameters of M3, and cell clustering and identification results from Scanpy were incorporated into Monocle3’s dimensionality reduction output. Trajectories were inferred using the *learn_graph* function, and root cells were specified based on prior knowledge to calculate pseudotimes for each cell.

### 2.10. Evaluation of Differentiation Potential

CytoTRACE (version 0.3.3) is a computational framework that estimates the differentiation potential of individual cells based on scRNA-seq data. CytoTRACE assigns a stemness score ranging from 0 to 1, where higher scores indicate less differentiated (more stem-like) cells. Differentiation scores were computed for each cell, allowing for the identification of starting points for cell differentiation and enabling the determination of pseudotime trajectories for root cells.

### 2.11. RNA Velocity Analysis

RNA velocity, which distinguishes between spliced and unspliced mRNA, was calculated using Velocyto (version 0.17.17) [21] and the STAR pipeline to process BAM files. Spliced and unspliced reads for each gene were counted, and results from all samples were pooled. RNA conversion rates for each cell were determined using scVelo (version 0.2.5) [22], and the future developmental trajectories of cells were projected onto the Monocle3 dimensionality reduction output. Classification followed the sub-class annotations obtained in earlier steps.

## 3. Results

### 3.1. snRNA-Seq Identified the Major Cell Types in Skeletal Muscle of Pigs

**Figure 1:**
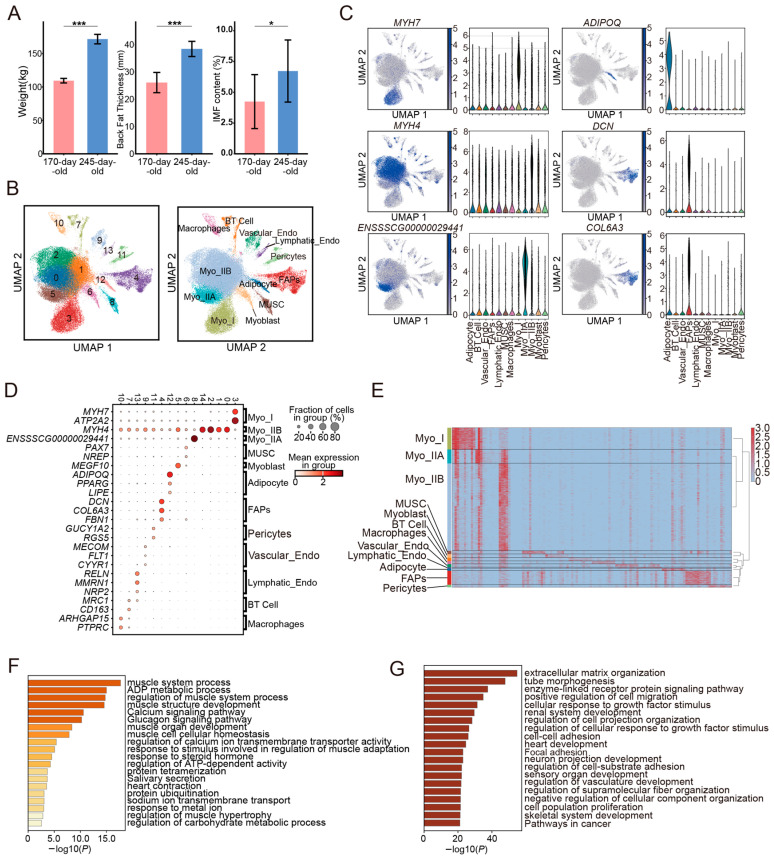
(**A**) Differences in body weight, backfat thickness, and intramuscular fat content of the longest dorsal muscle in 170-day-old and 245-day-old pigs (*n* = 2; *** indicates *p* < 0.001, * indicates *p* < 0.05). (**B**) Results of cell nuclear population clustering and annotation. (**C**) Main marker genes and expression levels of cell types in a violin plot. (**D**) Expression of marker genes in different cell clusters. (**E**) Heatmap of the top 10 differentially expressed genes across different cell types. (**F**) Myofiber enrichment pathway results. (**G**) FAP enrichment pathway results.

We observed a significant increase in body weight and subcutaneous fat in 245-day-old pigs compared with 170-day-old pigs (Figure 1A). Using sampling and soxhlet extraction of the anterior end of the dorsal longest muscle of pigs, we found a significant increase in IMF content in skeletal muscle of 245-day-old pigs. At the same time, we observed the results of stained sections of this area and found that fat content within skeletal muscle was significantly increased in 245-day-old pigs (Supplementary Figure S1B). In addition, 245-day-old pigs had thicker muscle fiber diameters and larger muscle fiber areas compared with 170-day-old pigs (Supplementary Figure S1A,C). To explore the cellular composition of pig skeletal muscle at the two ages, we applied snRNA-seq technology to isolate nuclei from the LDM of 170-day-old and 245-day-old pigs (*n* = 2). A total of 67,556 nuclei with a median UMI count of 490 and a median of 299 genes per nucleus were evaluated (Supplementary Table S1). After strict quality control, we retained 56,072 high-quality nuclei with less than 5% mitochondrial gene expression for subsequent analysis. These nuclei had a median UMI count of 509 and a median of 313 genes per nucleus, including 35,126 nuclei from 170-day-old pigs and 20,946 nuclei from 245-day-old pigs (Supplementary Table S2).

We removed batch effects due to sequencing technology using the Harmony method, followed by dimensionality reduction and unsupervised clustering of the high-quality nuclei. This resulted in a total of 15 initial cell clusters. Differential expression analysis and cell molecular marker genes allowed us to combine clusters expressing the same markers, identifying 12 major cell nuclear types in pig skeletal muscle (Figure 1B,D), including three types of myonuclei belonging to multinucleated muscle fibers (type I muscle fibers (Myo_I), type IIA muscle fibers (Myo_IIA), and type IIB muscle fibers (Myo_IIB)), muscle satellite cells (MUSCs), myoblasts, fibro/adipogenic progenitor cells (FAPs), adipocytes, pericytes, two types of endothelial cells (vascular endothelial cells and lymphatic endothelial cells), B cells and T cells (BT Cell) and macrophages (Figure 1C and Figure S2). Each cell type was characterized by its unique marker genes, and each had specific highly expressed differential genes (Figure 1E; Supplementary Table S3). Among all cell types, myofibers comprised the highest proportion (76.99%), while adipocytes represented only 1.46%, and fibro/adipogenic progenitor cells accounted for 8.73%. The remaining nine cell types made up 12.82%.

Gene Ontology (GO) and KEGG pathway enrichment analysis of differentially expressed genes (DEGs) for each cell type validated their respective characteristics and functions (Supplementary Figure S3). For instance, genes highly expressed in myofibers were significantly enriched in pathways such as muscle system processes and muscle structure development (Figure 1F). Notably, genes specifically expressed in type I myofibers were more significantly enriched in oxidative energy metabolism pathways, such as the tricarboxylic acid cycle, consistent with the oxidative nature of type I muscle fibers. Additionally, the enrichment results for other cell types were also in line with our annotations, such as the significant enrichment of tube morphogenesis pathways in FAPs and lipid metabolism pathways in adipocytes (Figure 1G).

### 3.2. Heterogeneity of Transcriptional Regulatory Networks Across Cell Types

**Figure 2:**
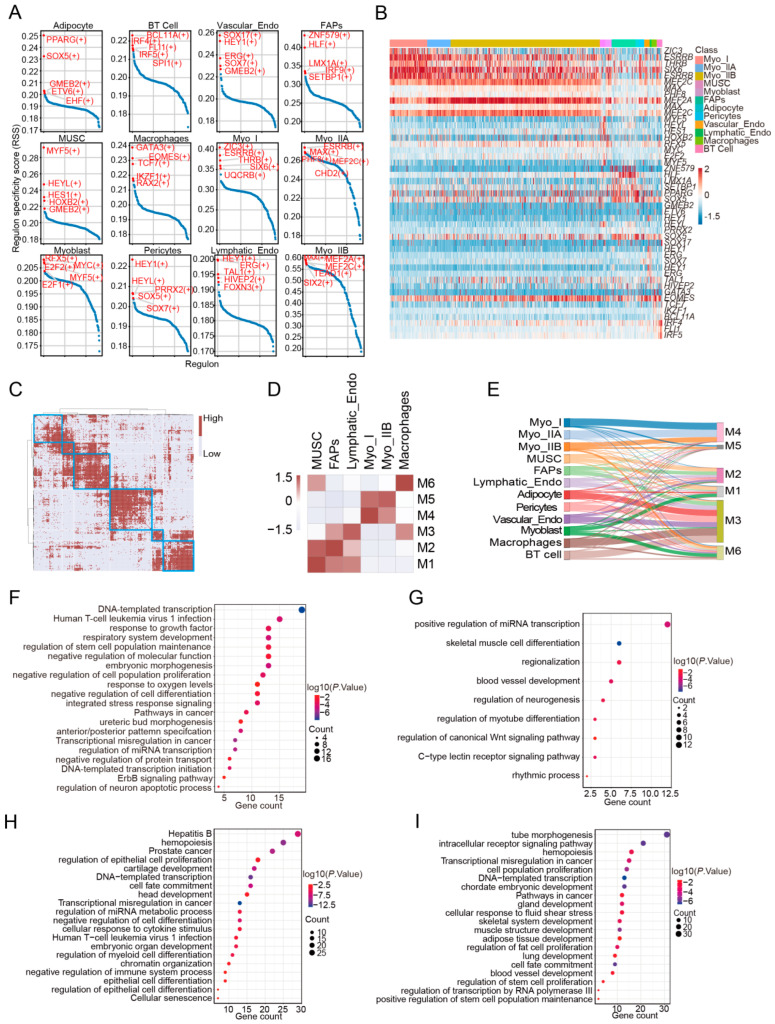
(**A**) The top five regulons for each cell type. Regulon specificity scores (RSS) of each annotated cell population. A point in a panel shows the RSS of one TF regulon. TF regulons are sorted by the RSSs in each cell type. The top five specific regulons are highlighted in red. (**B**) The expression activity of the top five regulons in each cell. (**C**) The unsupervised clustering results of the CSI matrix. Heatmap displays clustered regulon modules based on the CSI matrix along with the included regulons being shown in the right, indicating whether a given regulon is specific to a cell type. Top five RSS for each cluster shown. (**D**) The correspondence between regulon modules and the cells with the highest average activity of regulons. (**E**) The top 20 regulons in each cell appear in different modules. (**F**–**I**) Pathway enrichment results for M1, M2, M3, and M4, respectively.

Cell type diversity is achieved through the expression of transcriptional regulators that govern cell type-specific gene expression networks. To identify important regulatory factors and gene regulatory networks specific to different cell types in porcine skeletal muscle, we used the SCENIC tool to infer the activity and specificity of regulons—collections of transcription factors and their target genes. SCENIC provides regulon specificity scores (RSS) to assess differences in regulon activity across different cell types. As expected, we found that each major cell type has its unique transcriptional regulatory network (Figure 2A). For example, PPARG, a known regulator of adipocytes, showed the highest RSS value in adipocytes [23,24,25]. *MYF5*, involved in multiple processes of skeletal muscle cell differentiation, emerged as the most specific transcription factor in MUSCs and myoblasts [26,27]. Additionally, the regulon activity heatmap revealed similar activities of specific transcription factors in different myofiber subtypes, suggesting that transcription factors are shared across the same cell class (Figure 2B). In summary, through the transcription factor regulatory network, we identified distinct gene expression signatures in major skeletal muscle cell types, orchestrated by specific combinations of transcriptional regulators that contribute to cell type recognition.

Next, to systematically summarize the potential regulatory patterns among transcription factors and further evaluate the similarities and differences in transcriptional regulatory networks, we calculated the connectivity specificity index (CSI) for each regulon. The CSI characterizes the similarities among different regulons. First, 256 regulons were subjected to unsupervised clustering according to their CSI and then artificially divided into six modules based on the clustering tree. Each module contained regulons with potential co-regulation (Figure 2C). We hypothesized that the identity of a particular cell population is primarily determined by the corresponding regulatory clusters. For these six regulon modules, we calculated the average regulon activity for each module in each cell type, showing which cell type had the highest average activity in each module. Our results showed that specific cell modules corresponded to particular cell types, with M1 and M2 activated in MUSCs and fibro/adipogenic progenitor cells (FAPs), and M4 and M5 more active in myofibers (Figure 2D,I). The enrichment results for GO and KEGG pathways indicated that transcription factors in M1 and M2 were heavily involved in the maintenance of cell stemness and differentiation, while MUSCs and FAPs are known precursors of mature myofibers and the mature fiber/fat components of skeletal muscle (Figure 2F,G). Among the cell types in skeletal muscle, MUSCs and FAPs are highly stem-like and involved in differentiation. These findings suggest that although different cell types have distinct differentiation endpoints, cells with differentiation potential may share a unified transcriptional regulatory network, and different cells may be regulated by the same transcription factors during differentiation.

Finally, we explored the distribution model of the top 20 cell-type-specific regulons for each cell type across the six modules, represented in a Sankey diagram (Figure 2E). Consistent with the previous results, we observed the enrichment of top regulons in myofibers within M4 and M5, while M1 and M2 contained many specific regulons for cells with higher stemness, such as MUSCs, myoblasts, and FAPs. Interestingly, M3 contained a significant number of specific regulons for immune cell types, along with transcription factors related to FAPs and adipocytes, suggesting that immune cells may influence the process of intramuscular fat deposition. As recently reported, macrophages impact the adipose-forming potential of intramuscular FAPs by stimulating SMAD signaling, and IL-4-polarized macrophages enhance intracellular lipid accumulation [28]. Coincidentally, we found members of the SMAD family in M3, and the M3 enrichment results also included the GO pathway in response to IL4 signaling (Figure 2H). These findings provide some support for our hypothesis.

### 3.3. Cell–Cell Communication Analysis Uncovered Cell Interactions in Skeletal Muscle Across Two Stages

**Figure 3:**
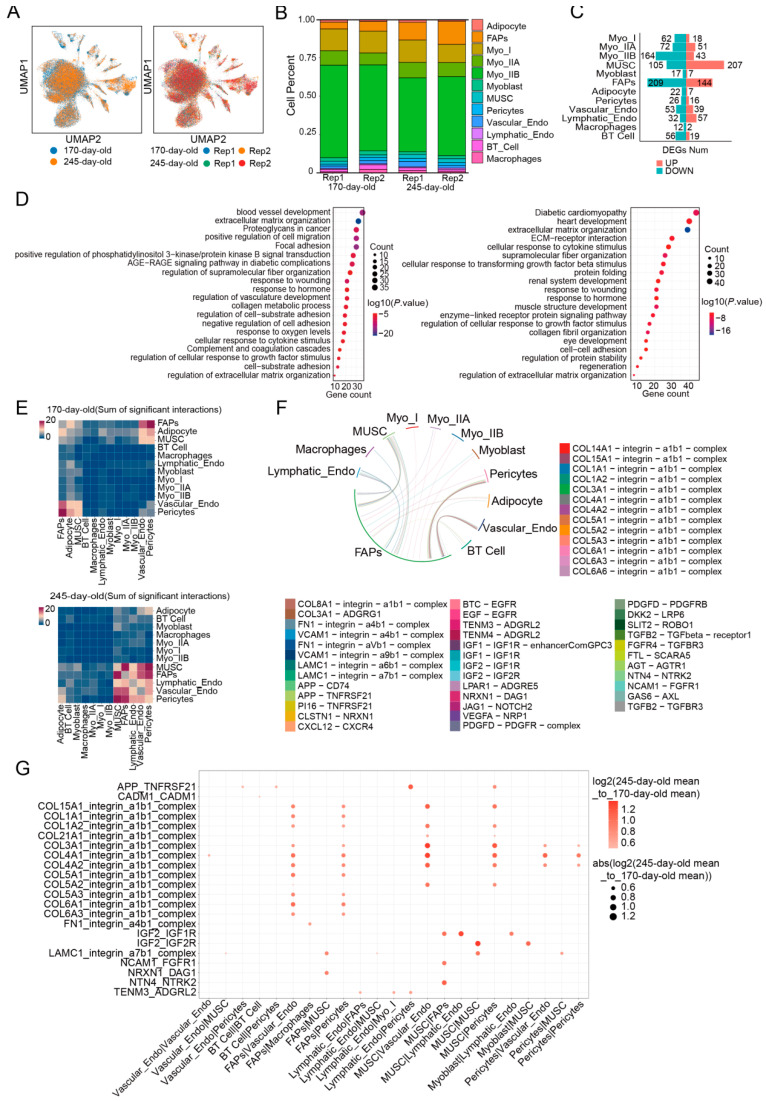
(**A**) Uniformity of cell types between two stages. (**B**) Cell composition of pigs at two stages. (**C**) Number of differentially expressed genes for each cell type between two stages (|logFC| > 1, *p.adj* < 0.05). (**D**) Enrichment results for differentially upregulated gene pathways in FAPs and MUSCs. (**E**) Number of cellular communications between two stages. (**F**) Violin plot of cell-to-cell communication between FAPs and other cells in the 245-day-old pigs. (**G**) Differences in expression levels of cell-to-cell communication between the two stages.

To investigate differences in skeletal muscle cell composition between the two stages, we first examined the cell types and their percentage composition in individuals of different ages. As expected, no new cell types appeared in the skeletal muscle of pigs during the normal growth phase, and the cell types at the two stages were consistent. Differences in cellular components were primarily reflected in changes in the percentage of cells (Figure 3A,B).

In the proportion of myofibers, we observed a slight decrease in myofiber content in 245-day-old pigs compared to 170-day-old pigs (170-day-old: 79.73%, 245-day-old: 72.39%). This trend was largely due to a reduction in the percentage of type IIB nuclei (170-day-old: 57.25%, 245-day-old: 50.32%), while the percentage of type IIA nuclei increased slightly (170-day-old: 8.24%, 245-day-old: 9.39%). Although adipocyte content showed little difference (170-day-old: 1.49%, 245-day-old: 1.41%), the percentage of FAPs was significantly different between the two stages. FAPs accounted for 13.95% of the total nuclei in 245-day-old pigs, more than double the percentage in 170-day-old pigs (5.63%).

Interestingly, we found a decrease in the proportion of myoblasts and, conversely, an increase in the proportion of MUSCs in 245-day-old pigs. As both quiescent and activated states of myosatellite cells, MUSCs and myoblasts are jointly involved in the growth and replenishment of myofibers. The observed differences in these cell components between the two stages may suggest that, as individuals mature, myosatellite cells transition from an activated state to a resting state, serving as “backup” precursor cells for myofiber regeneration during injury repair [29]. Moreover, we observed that FAPs and MUSCs exhibited the most differential gene expression between the two stages. Genes significantly upregulated in MUSCs and FAPs from 245-day-old pigs were involved in activating growth factor-stimulated pathways, indicating that growth-related processes in 245-day-old pigs are upregulated in the precursor cells (Figure 3C,D; Supplementary Table S4).

In cases where cell types are nearly identical, differences between tissues or individuals often arise from changes in cell communication. Thus, we explored the ligand–receptor interactions between cells from pigs of different ages using CellphoneDB. We observed that cell–cell communication occurred significantly more frequently in 245-day-old pigs (Figure 3E). In 170-day-old pigs, communication was mainly observed between FAPs, adipocytes, MUSCs, pericytes, and vascular endothelial cells. In contrast, in 245-day-old pigs, the number of interactions involving adipocytes decreased, while communication between MUSCs and FAPs remained high, with more ligand–receptor interactions occurring between these two cell types (Figure 3E).

Comparative analyses of ligand–receptor pairs with significant interactions between the two stages revealed that the strength of these interactions was consistently upregulated in 245-day-old pigs. (Figure 3G). A large number of collagen and integrin family interactions were observed in the intercellular communication of FAPs, and the strength of these interactions was enhanced in 245-day-old pigs (Figure 3F,G). This type of communication is an important mechanism for fibrillation in FAPs in skeletal muscle and supports the morphological structure of myofibers [30]. Additionally, in MUSC communication, we observed a stronger IGF2–IGF1R interaction in 245-day-old pigs. Several studies have shown that the interaction between this ligand–receptor pair promotes muscle growth and development [31].

### 3.4. Characterization of FAPs in Skeletal Muscle by Clustering and Pseudotime Analysis

**Figure 4:**
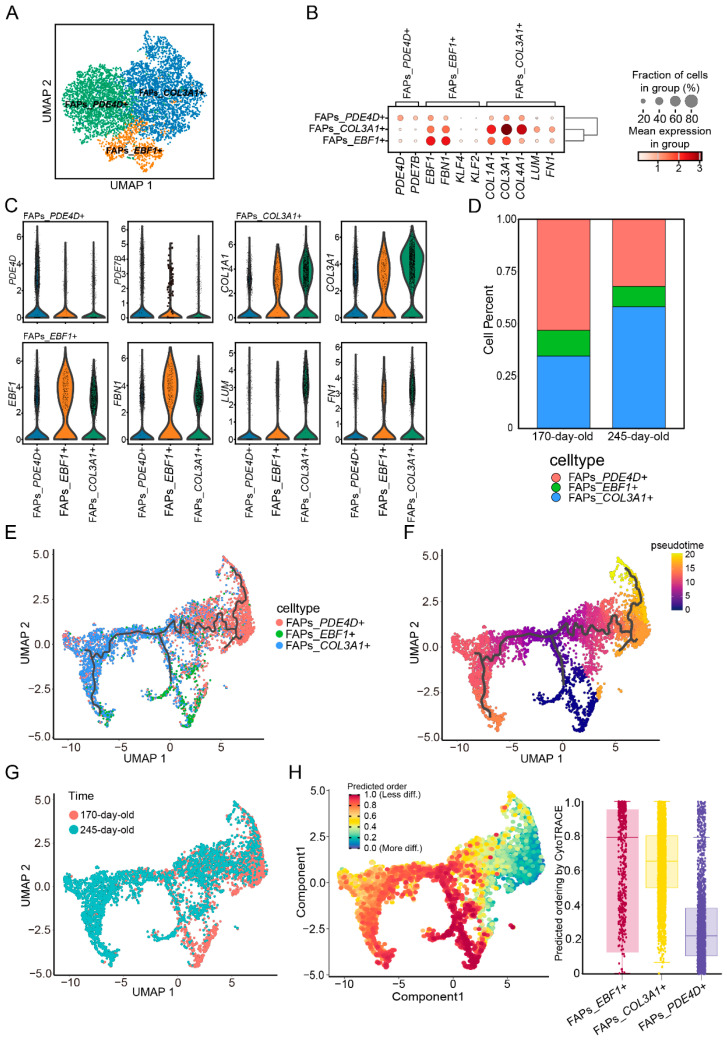
(**A**) Original clustering results and cell annotation of FAP cells. (**B**) Expression of marker genes in different subtypes. (**C**) Violin plot of marker gene expression in major cell types. (**D**) Proportion of FAP subtypes at different developmental stages. (**E**) Pseudotime trajectory of FAPs. (**F**) Developmental pseudotime of FAP subtypes. (**G**) Distribution of FAP cells along the pseudotime trajectory in individuals at different developmental stages. (**H**) Differences in differentiation levels of FAP subtypes.

We observed that FAP cells exhibited the greatest differences in terms of cellular components, gene expression, and cellular communication between the two growth stages. Therefore, we further explored the nuclei of FAPs within the muscles of pigs at these two different stages. Initially, 4897 FAP nuclei were extracted and analyzed using dimensionality reduction and cluster analysis. Combined with differential expression analysis, the FAPs were divided into three subclasses (Figure 4A). These included the FAPs_*PDE4D*^+^ subpopulation, which highly expresses *PDE4D* and *PDE7B* genes. This subpopulation has been reported to resemble adipocytes and tends to be enriched in individuals with high adiposity [9]. The FAPs_*EBF1*^+^ subpopulation highly expresses *EBF1* and *KLF4* genes, which are associated with early adipocyte formation in adipose tissue. Additionally, the FAPs_*COL3A1*^+^ cell types, which highly express fibrotic cell formation genes like *COL3A1*, were identified (Figure 4B). The bubble and violin diagrams illustrate the expression of these molecular marker genes across the three FAP subclasses (Figure 4C).

To further investigate the differentiation process of FAPs in skeletal muscle development, we analyzed the continuous cell state of FAPs using pseudotime trajectory analysis. The ordering of cells in pseudotime revealed a major trajectory for most FAPs, which split into two endpoints, each pointing towards one of the two FAP subclasses (Figure 4E). To determine the starting point of the pseudotime trajectory, we used Cytotrace to calculate the differentiation stemness of the three FAP subclasses. The results showed that FAPs_*EBF1*^+^ cells were the most stem-like, with the lowest differentiation degree, followed by FAPs_*COL3A1*^+^ cells, which possess the potential to differentiate into fibrocytes. FAPs_*PDE4D*^+^ cells had the highest differentiation degree (Figure 4H). This indicates that FAPs_*EBF1*^+^ cells in skeletal muscle are high-stemness pluripotent progenitor cells. Along the pseudotemporal trajectory, FAP nuclei differentiated from the FAPs_*EBF1*^+^ subclass into two branches, subsequently differentiating into the FAPs_*COL3A1*^+^ and FAPs_*PDE4D*^+^ subclasses, respectively. The pseudotemporal maps showed that the FAPs_*PDE4D*^+^ subclass was at the latest stage of FAP differentiation, with a later differentiation rate compared to the FAPs_*COL3A1*^+^ subclass, which was the most maturely differentiated subclass among FAPs (Figure 4F).

Notably, we observed a significantly higher proportion of the FAPs_*COL3A1*^+^ subpopulation in the skeletal muscle of 245-day-old pigs compared to 170-day-old pigs (170-day-old: 34.62%, 245-day-old: 58.13%). In contrast, the proportion of FAPs_*EBF1*^+^ cells decreased in 245-day-old pigs (Figure 4D). This suggests that FAP cells in 245-day-old pigs are closer to completing the differentiation process, indicating an end stage of differentiation. This trend aligns with previously reported decreases in the proportion of FAP cells resembling FAPs_*EBF1*^+^ subclasses in obese mouse adipose tissue [32]. Moreover, our differentiation trajectory analysis clearly captured the nuclear transitions at different stages. The results showed that the majority of FAPs cells in 245-day-old pigs were distributed at the end of the differentiation trajectory, although a small number of nascent FAP cells were still present at the root of the pseudotime trajectory. In contrast, FAP cells from 170-day-old pigs were primarily located at the beginning of the trajectory. Consistent with the cell percentage results, FAPs in 245-day-old pigs were more likely to follow the differentiation pathway of the FAPs_*COL3A1*^+^ subclass, appearing later in the differentiation trajectory compared to 170-day-old pigs. In contrast, the FAPs_*PDE4D*^+^ subclass in 170-day-old pigs tended to cluster at the end of the trajectory (Figure 4G).

### 3.5. Differentiation Trajectory of IMF in Pig Skeletal Muscle

**Figure 5:**
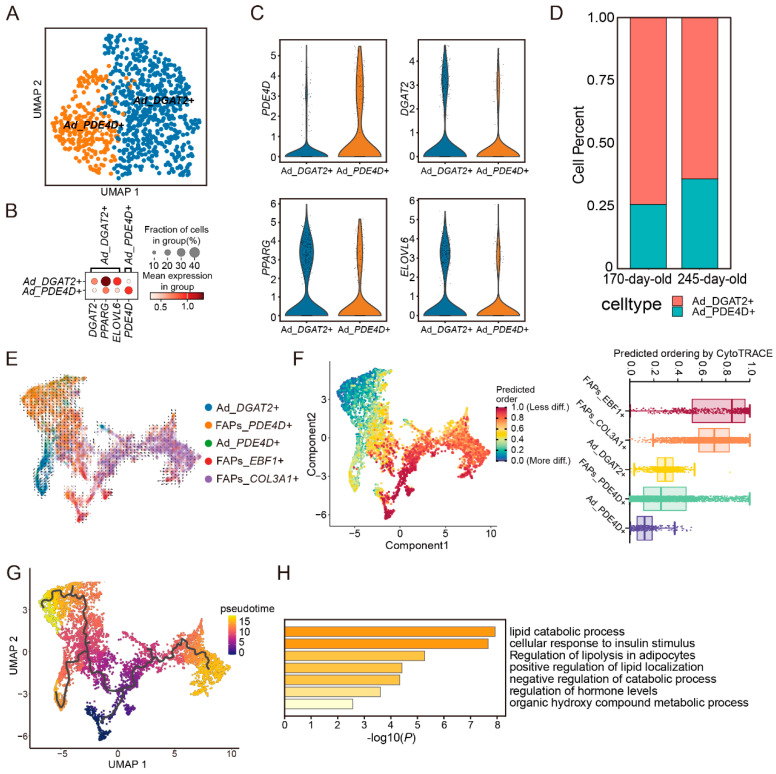
(**A**) Original clustering results and cell annotation of adipocyte cells. (**B**) Expression of marker genes in different subtypes. (**C**) Violin plot of marker gene expression in major cell types. (**D**) Proportion of adipocyte subtypes at different developmental stages. (**E**) RNA velocity plot of FAPs and adipocyte cells. (**F**) Differences in differentiation levels between FAPs and adipocyte cells. (**G**) Distribution of FAPs and adipocyte cells along the pseudotime trajectory in individuals at different developmental stages. (**H**) Pathway enrichment analysis of Ad_*DGAT2*^+^ adipocyte cell-specific expressed genes.

Previous studies have demonstrated that FAPs have the potential to differentiate into intramuscular fat (IMF) cells and are a primary source of these cells [33,34,35]. In this study, we aimed to investigate the effects of changes in FAPs on intramuscular adipocytes in the skeletal muscle of pigs at two different growth stages. To this end, we performed a subpopulation analysis of adipocytes, classifying them into two groups: the Ad_*DGAT2*^+^ subpopulation and the Ad_*PDE4D*^+^ subpopulation, based on their distinct gene expression profiles. The Ad_*PDE4D*^+^ subpopulation was more prevalent in 245-day-old pigs (Figure 5A,D). *DGAT2* has been identified as a key gene regulating the rate of triacylglycerol (TAG) synthesis in multiple species, with TAG being the main component of IMF. Given the high expression of the *PPARG* gene, it is reasonable to conclude that the Ad_*DGAT2*^+^ subclass represents a population of mature adipocytes (Figure 5B,C) [36,37,38].

RNA velocity and pseudotime analyses, which integrate IMF-associated cells—namely FAPs—revealed the formation process of IMF deposits in skeletal muscle. As expected, the differentiation trajectory showed that mature adipocytes were located at the end of the trajectory from FAPs to adipocytes (Figure 5E,F). RNA velocity analysis indicated the future direction of cells based on the arrow’s pointing and length. Our results suggest that mRNA expression in the Ad_*DGAT2*^+^ subclass undergoes rapid turnover (Figure 5E). Functional enrichment analysis showed that genes highly expressed in the *DGAT2* subpopulation were significantly enriched in lipid metabolism-related pathways (Figure 5H), indicating that these adipocytes represent an active metabolic state for mature fat in the IMF.

In the differentiation trajectory map, we observed two trajectories originating from the adipose precursor subclass of FAP cells, pointing to two distinct adipocyte subclasses (Figure 5G). Notably, Ad_*DGAT2*^+^ adipocytes differentiate from a portion of the FAPs_*EBF1*^+^ subclass, while the remainder of the FAPs_*EBF1*^+^ cells advance towards the FAPs_*PDE4D*^+^ subclass and eventually differentiate into Ad_*PDE4D*^+^ adipocytes [12]. We also observed that the Ad_*PDE4D*^+^ subclass differentiated later than the Ad_*DGAT2*^+^ subclass (Figure 5F,G). Previous studies have shown that *DGAT2* subclasses are the primary cell type responsible for differences in intramuscular adiposity, and the *PDE4D* gene plays an anti-adipolytic role in adipocytes [9,39,40]. We speculate that, in porcine skeletal muscle, after FAPs differentiate into the mature adipocyte subclass with high *DGAT2* expression, some of the FAP_*PDE4D*^+^ cells differentiate into the *PDE4D* adipocyte subclass to protect the mature adipocytes from catabolism. The greater number of *PDE4D* cells in 245-day-old pigs suggests that anti-lipolytic processes facilitate IMF deposition in skeletal muscle.

## 4. Discussion

We sought to explore changes in porcine skeletal muscle at the cellular level between two stages of growth and development. Our analysis identified 12 distinct cell types in the LDM of pigs, with differences in their proportions between the two stages. This suggests that the differences between growth stages are primarily reflected in cellular composition rather than in distinct cell types. Except for myofibers, FAPs represented the highest percentage of cells in porcine skeletal muscle. Notably, we observed a decrease in the total proportion of myofibers, while the proportion of FAPs was higher in 245-day-old pigs compared to 170-day-old pigs.

FAPs have been shown to be multifunctional progenitor cells capable of differentiating into fibroblasts and adipocytes, both of which are key contributors to the intramuscular connective tissue [41,42,43,44,45,46,47]. FAPs interact with MUSCs in skeletal muscle, promoting differentiation and driving muscle fiber formation [35,43]. The transcriptional regulatory network shared by FAPs and MUSCs, as identified in our study, may be one of the mechanisms by which these cells co-regulate myofiber development. Our results suggest that, in 245-day-old pigs, FAPs are more inclined towards fibroblast differentiation, resembling the increased fibrosis observed in skeletal muscle in elderly humans. High expression of collagen in fibroblasts enhances the toughness of porcine muscle and improves the tenderness and texture of pork, whereas excessive collagen deposition in the human body is often associated with pathological conditions that inhibit the regenerative capacity of myoblasts, leading to muscle dystrophy and atrophy [48,49].

Triacylglycerol is the main component of intermuscular fat, and the expression of diacylglycerol o-acyltransferase 2 (*DGAT2*) regulates the rate of TAG synthesis [50,51]. The *PDE4D* gene also plays a crucial role in regulating fat deposition and preventing its breakdown [39]. Our findings suggest that adipocytes in skeletal muscle differentiate from different subpopulations of FAPs. The Ad_*PDE4D*^+^ subpopulation of adipocytes differentiated later than the Ad_*DGAT2*^+^ subpopulation, with a higher percentage of Ad_*PDE4D*^+^ adipocytes observed in 245-day-old pigs. We hypothesize that the Ad_*PDED4*^+^ subclass may regulate the Ad_*DGAT2*^+^ subclass of mature adipocytes, promoting skeletal muscle fat deposition and preventing excessive fat breakdown. However, the exact mechanism requires further investigation.

## 5. Conclusions

In conclusion, our study provides a cellular atlas of porcine skeletal muscle at different growth stages, highlighting differences in transcriptional regulation across various cell types. We also describe changes in skeletal muscle cellular composition and communication at different growth stages. Furthermore, we have constructed cellular composition and differentiation trajectories of skeletal muscle adipocytes and their precursor cells across growth stages. Our results offer insights into the cellular-level differences in skeletal muscle at different stages of growth in pigs, providing a theoretical foundation for understanding the regulation of IMF deposition during growth and development.

## Data Availability

The snRNA-seq data of porcine skeletal muscle generated in this study have been deposited in the NCBI’s GEO under accession code GSE279877. The code used for the single-cell analysis mentioned in the article is publicly available on GitHub at “https://github.com/SICAU-ChenZiyu/skeletal-muscle-at-different-growth-stages”.

## Supplementary Materials

The following supporting information can be downloaded at: https://www.mdpi.com/article/10.3390/cells14010037/s1, Figure S1: Skeletal muscle phenotypes in pigs of different ages; Figure S2: Marker genes and their expression in each cell types; Figure S3: Pathway enrichment results for highly expressed genes in various cell types; Table S1: snRNA-seq mapping information; Table S2: high-quality nucleus of each samples; Table S3: Differential expression of genes in major cell types; Table S4: Differentially expressed genes (245-day-old pigs vs. 170-day-old pigs).
