# Supplementary figures and images for: Single-Nucleus RNA Sequencing Reveals Cellular Transcriptome Features at Different Growth Stages in Porcine Skeletal Muscle
Source: Cells. 2025 Jan 2;14(1):37. doi: 10.3390/cells14010037 (PMC11720419; doi:10.3390/cells14010037)

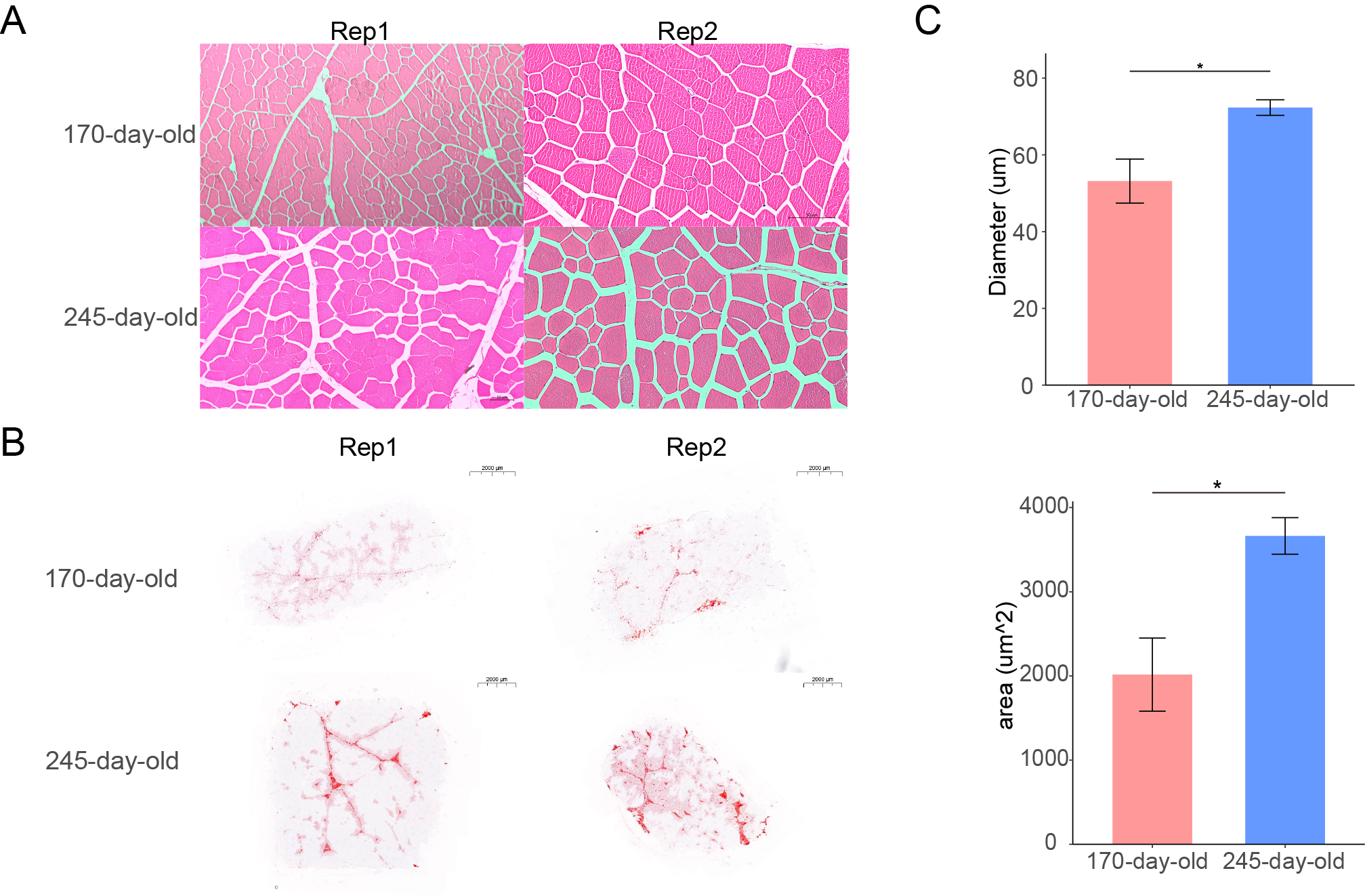

Supplement: Supplementary file 1 [file cells-14-00037-s001.zip › cells-3393369-supplementary/Supplementary Figure S1.png]

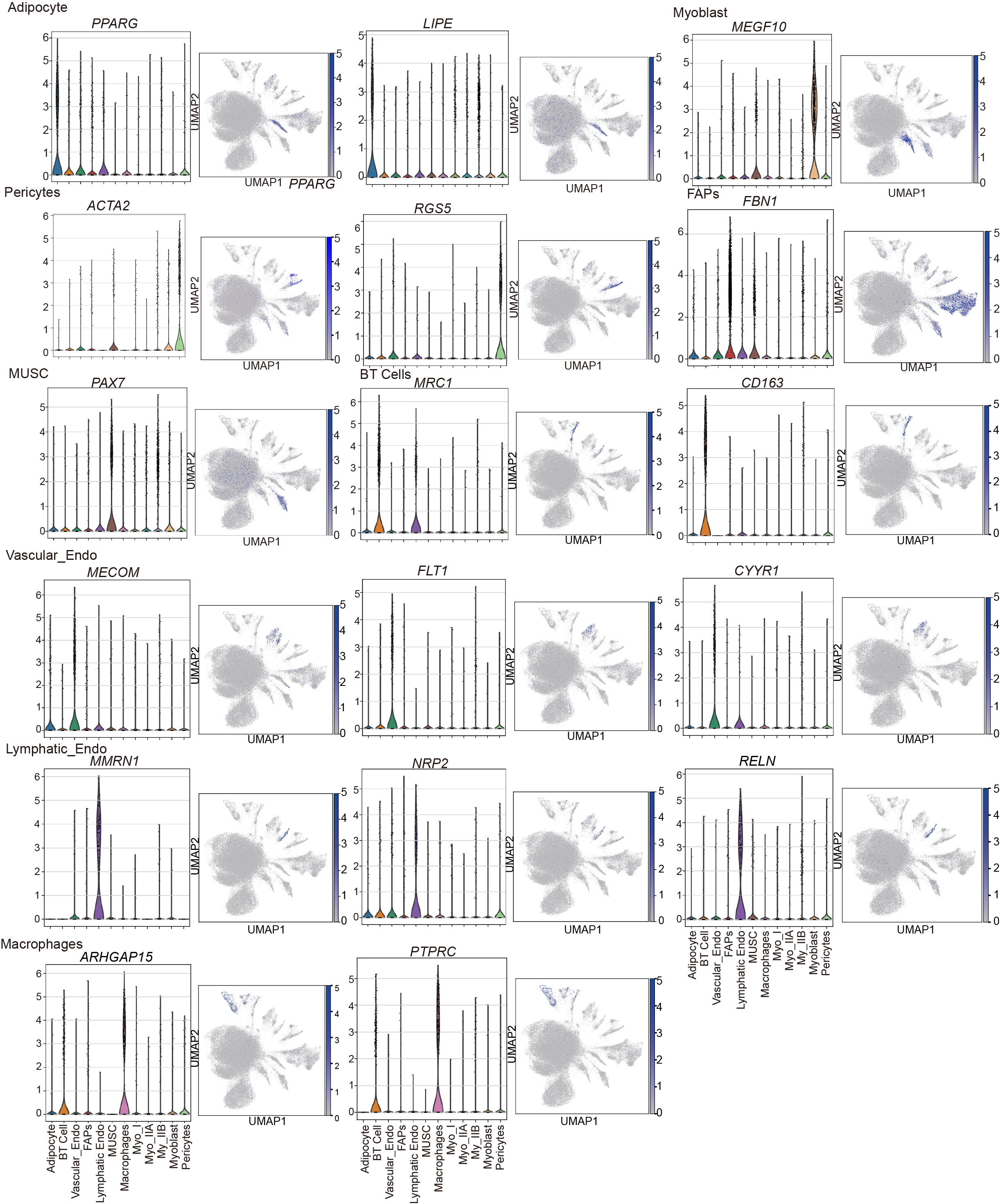

Supplement: Supplementary file 1 [file cells-14-00037-s001.zip › cells-3393369-supplementary/Supplementary Figure S2.png]

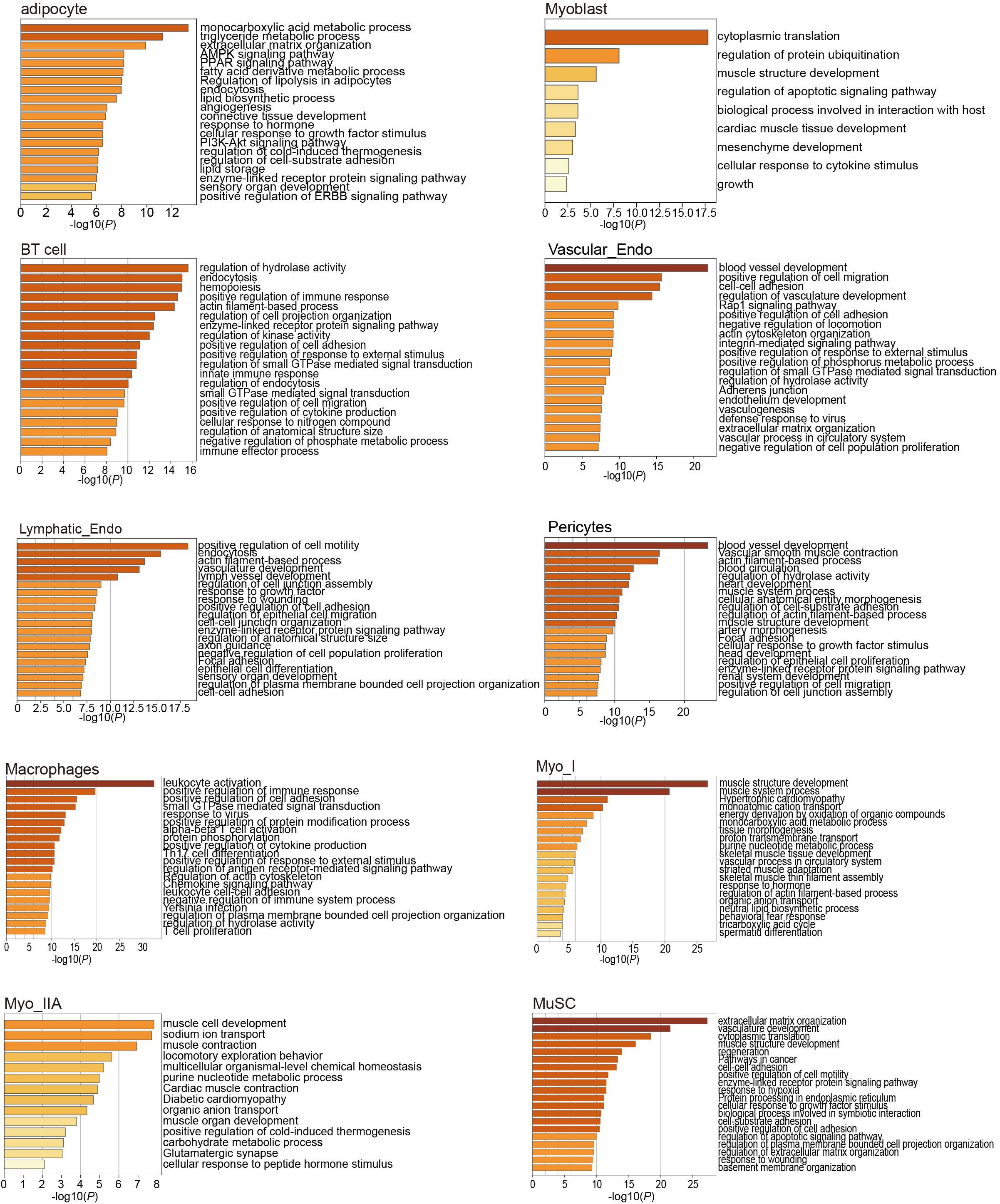

Supplement: Supplementary file 1 [file cells-14-00037-s001.zip › cells-3393369-supplementary/Supplementary Figure S3.png]
